# Portopulmonary hypertension: Success with combined medical treatment

**DOI:** 10.1002/rcr2.1114

**Published:** 2023-03-16

**Authors:** María Hernández, Violeta Esteban, Paloma Ruiz, Ignacio Boira, Phillip Wickmann, José N. Sancho‐Chust, Joan Gil

**Affiliations:** ^1^ Department of Respiratory Medicine Hospital Universitario de San Juan Alicante Spain; ^2^ Department of Respiratory Medicine Hospital General Universitario de Alicante Alicante Spain; ^3^ Department of Internal Medicine Hospital Universitario de San Juan Alicante Spain

**Keywords:** hepatitis C, portal hypertension, portopulmonary hypertension, pulmonary hypertension, shistosomiasis

## Abstract

Portopulmonary hypertension is an uncommon disease associated with high morbidity and mortality, so its early diagnosis and treatment are essential. We report here the case of a 57‐year‐old man with portopulmonary hypertension caused by chronic hepatosplenic schistosomiasis and also liver cirrhosis due to hepatitis C and alcoholism. As well as treating both diseases, portopulmonary hypertension was successfully managed with tadalafil and macitentan as maintenance therapy. This case reminds clinicians that pulmonary hypertension can be multifactorial, a good diagnosis and a multidisciplinary treatment can lead to improved prognosis.

## Graphical abstract

We present a patient with portopulmonary hypertension (POPH) successfully treated with the combination of tadalafil and macitentan.

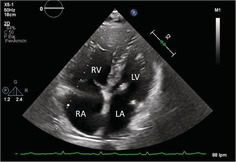

## INTRODUCTION

Portopulmonary hypertension (POPH) is a serious pulmonary vascular complication of cirrhotic and noncirrhotic portal hypertension, that only appears in 2%–6% of patients with portal hypertension and associates significant mortality.^1^, ^2^ There is little evidence supporting the use of pulmonary arterial hypertension (PAH) therapies in patients with POPH because the overwhelming majority of clinical trials evaluating PAH‐specific therapies have excluded POPH patients.^1^, ^2^ PORTICO was the first randomized controlled trial of PAH therapy specifically done in patients with portopulmonary hypertension.^3^

We present a patient with POPH successfully treated with the combination of tadalafil and macitentan.

## CASE REPORT

A 57‐year‐old man, who quit smoking in February 2020, with a cumulative smoking of 40 pack‐years, and former alcoholic of six *standard alcoholic drinks*, with history of arterial hypertension, hiatal hernia, insomnia and *Chronic Obstructive Pulmonary Disease* (COPD). He had a forced expiratory volume in the first second (FEV1) of 69% predicted, he did not have frequent exacerbations and was taking indacaterol/glycopyrronium (85 mcg and 43 mcg) as inhaled combination therapy. He attends the department of respiratory medicine, due to worsening dyspnea, with a functional status WHO FC‐II. He worked as a gemologist, travelling usually to South America and central Africa, the last trip being 3 months ago.

**FIGURE 1:**
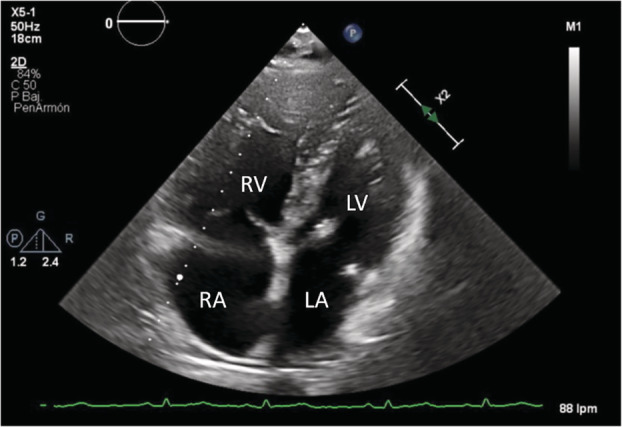
Transthoracic echocardiogram. There is marked dilatation of the right chambers, with displacement of the interventricular septum to the left. (RA, right atrium; RV, right ventricle; LA, left atrium; LV, left ventricle)

**FIGURE 2:**
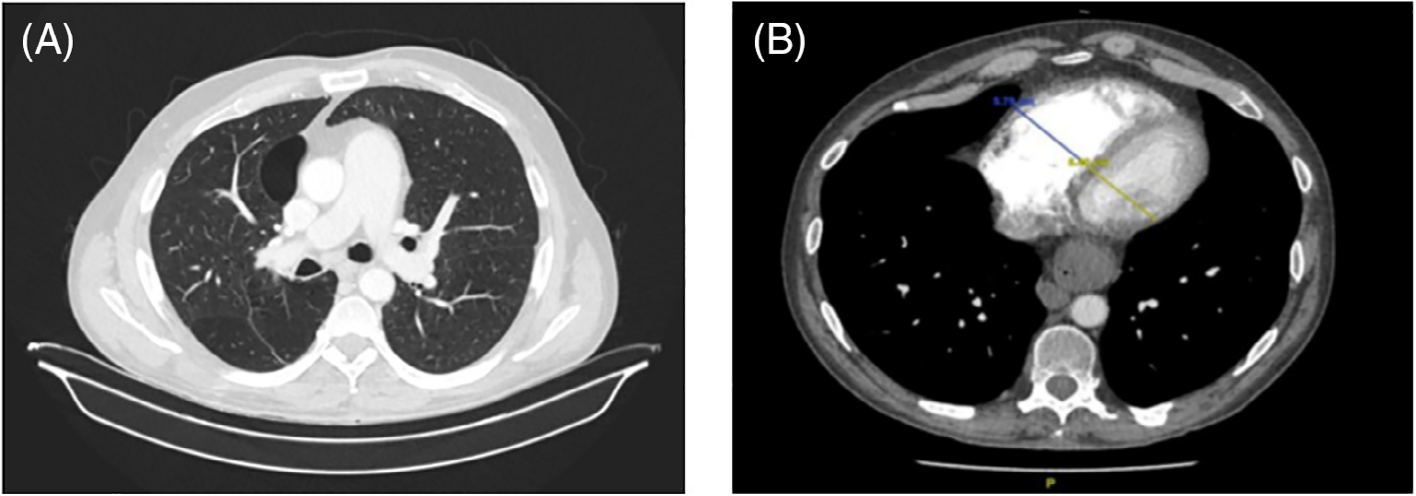
(A) Significant centrolobular and paraseptal emphysema with biapical predominance, as well as dilated pulmonary artery cone (38 mm). (B) Dilatation of right cavities with displacement of the interventricular septum to the left

**FIGURE 3:**
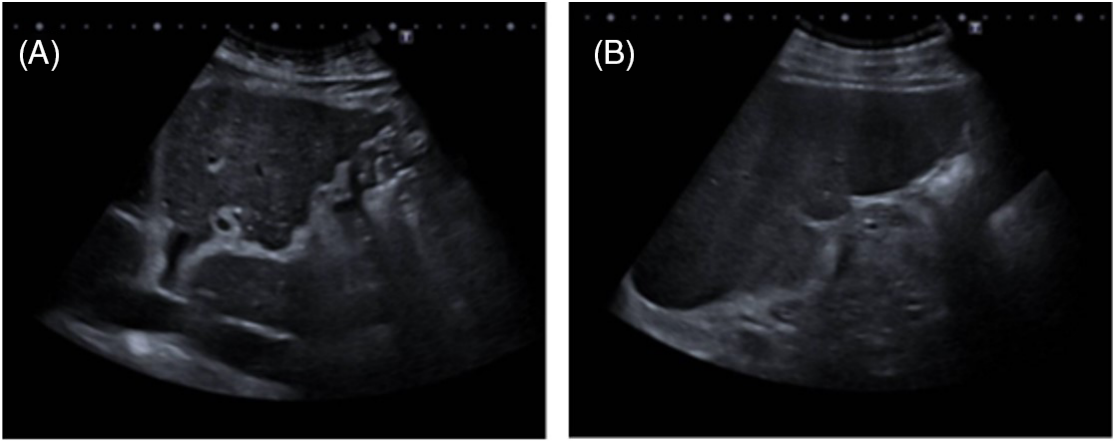
Liver ultrasound. (A) Cirrhotic liver with enlargement of the left lobe and periportal fibrosis. (B) Splenomegaly of 17 cm in longitudinal diameter

At first, we decided to complete the study with a transthoracic echocardiography bubble study that evidenced dilatation and hypertrophy of the right chambers, estimating pulmonary artery systolic pressure (PAsP) around 65 mmHg, with preserved right ventricular ejection fraction at that time; TAPSE was 24 mm; and left ventricular ejection fraction was normal, without relevant valvulopathies or intracardiac shunts (Figure 1). Lung function test showed moderate obstruction (FEV1 69% predicted) and a severe decrease of the carbon monoxide diffusing capacity (DLCO 33% predicted). The distance covered in the 6‐min walk test (6MWT) was 150 m (27% predicted) with severe desaturation (nadir SpO_2_ of 88%). Chest computed tomography (CT) showed significant centrolobulillar and paraseptal emphysema which mainly affected *the upper lobes*; dilated pulmonary artery, measuring 38 mm in diameter, as well as dilated right chambers (Figure 2). In the presence of probable severe pulmonary hypertension, a complete blood test was performed with autoimmunity and serology for *Schistosoma*, hepatitis B, hepatitis C virus, and HIV. Abdominal ultrasound, respiratory polygraphy, ventilation/perfusion scintigraphy and right heart catheterization (RHC) were too performed. Laboratory findings showed platelets 87 10e9/L, AST 62U/L, Gamma GT 181U/L and a C‐reactive protein of 2.37 mg/dL, with negative autoimmunity. Abdominal ultrasound elucidated a cirrhotic liver with enlargement of the left lobe and periportal fibrosis and splenomegaly (Figure 3), while ventilation/perfusion scintigraphy concluded a low probability of pulmonary thromboembolism. Sleep study confirmed mild sleep apnea syndrome. Positive IgG serology obtained for hepatitis C and *Schistosoma, with a viral load RNA HCV of 1.700.000 UI/mL*. The patient was referred to the internal medicine department where hepatitis C was treated with velpatasvir + sofosbuvir 100/400 mg during 12 weeks and schistosomiasis was treated with praziquantel 3600 mg in a single dose. The RHC confirmed severe pre‐capillary pulmonary hypertension, with a mean PAP of 52 mmHg, pulmonary arterial wedge pressure of 12 mmHg, atrial right pressure of 9 mmHg, cardiac outpout of 7.77 L/min, cardiac index of 3.61 L/min/m^2^, pulmonary vascular resistance (PVR) of 4.63 Wood units and the acute vasoreactivity test was negative. Treatment with sidenafil 20 mg/8 h was started, a continuous flow portable oxygen device at 2 L/min was also prescribed and bosentan 62.5 mg/12 h was added 3 months later.

On follow‐up, the patient had no improvement despite treatment, he had a syncopal episode and functional status WHO FC‐III, with NT‐proBNP at 601 pg/mL; therefore, it was decided to refer him to the pulmonary hypertension center of reference. The patient was admitted for iron deficiency severe anaemia with haemoglobin at 5.4 mg/dL, and a new CT scan evidenced gastric and splenic varices, as well as *enlarged spleen* and *dilatation* of the *portal vein*. An endoscopic ultrasound was requested obtaining a portal pressure gradient of 23 mmHg and large oesophageal varices, all confirming the existence of portal hypertension. Finally, all these results confirmed the diagnosis of Portopulmonary Hypertension (POPH), with severe hemodynamic involvement, so the treatment was changed 1 year and 3 months from the beginning to tadalafil 40 mg/24 h + macitentan 10 mg/24 h.

After 4 months of treatment adjustment, the patient improved to WHO FC‐I, NT‐proBNP decreased to 61 pg/mL and the distance covered in the 6MWT improved to 498 metres without desaturation (SpO_2_ > 90%).

## DISCUSSION

POPH is defined as pulmonary hypertension that occurs as a consequence of portal hypertension with or without liver disease, and is a subset of group 1 pulmonary hypertension.^1^, ^2^ In the presence of documented portal hypertension, is defined according to the following hemodynamic data obtained during a RHC: (a) mean pulmonary artery pressure ≥20 mmHg; (b) PVR ≥2 Wood units; and (c) pulmonary artery wedge pressure ≤15 mmHg.^1^ In addition, the severity of POPH is defined based on the mPAP as follows: mild (20–34 mmHg), moderate (35–44 mmHg), and severe (≥45 mmHg). The vast majority of cases of POPH appear in patients with portal hypertension related to cirrhosis, but non‐cirrhotic causes of portal hypertension are also important contributors (portal vein thrombosis, granulomatous disease, auto‐immune disorders causing hepatitis, drug reactions, congenital abnormalities, and infections like chronic hepatosplenic shistosomiasis).^2^ Only 2%–6% of patients with portal hypertension also develop a pulmonary arterial hypertension.^2^ In patients with POPH who had cirrhosis, pulmonary vascular bed is exposed to multiple circulating growth factors, neurohormone levels, and cytokine levels. Of particular importance are the increased levels of circulating endothelin‐1, a potent vasoconstrictor and facilitator of smooth muscle proliferation. On the other hand, pulmonary vascular bed may be deficient in local nitric oxide effect, a potent vasodilator.^2^ Chronic hepatosplenic schistosomiasis is another cause of POPH, usually due to *Schistosoma mansoni*.^4^ Portal hypertension results in portocaval shunts, increased pulmonary flow with resultant endothelial dysfunction, and subsequent pulmonary artery hypertension.^4^ Other hypothesis is that the passage of the parasite or its eggs through the lungs induces endothelial dysfunction and release of inflammatory mediators, with formation of plexiform lesions, but with a slower rate of progression.^4^ Only 7.7% of patients with hepatosplenic shistosomiasis will develop pulmonary hypertension.^4^ The diagnosis of hepatosplenic schistosomiasis is based largely on a history of environmental exposure to the parasite and the identification of the parasite eggs in stool examination or on rectal biopsy. Also abdominal ultrasonographic findings of periportal fibrosis and enlargement of the left lobe of the liver and serological tests in non‐endemic areas are useful for stablishing the diagnosis. Anthelmintics help to stop disease progression and there may be some clinical improvement.^4^

Poor correlation exists between the severity of POPH and the degree of liver dysfunction and with hepatic venous pressure gradient.^2^ Patients with POPH had poorer survival rates compared with patients with idiopathic or drug‐induced pulmonary hypertension (only 40% of patients after 5‐year time of diagnosis), and this could be because, in POPH, death can occur due to right failure or digestive complications (upper gastrointestinal bleeding, sepsis or hepatocellular carcinoma).^2^ The immediate goal in the treatment of POPH is to improve pulmonary hemodynamics by reducing the obstruction to pulmonary artery flow, improving and/or normalizing right ventricle (RV) function.^1^, ^2^ This can be accomplished by medications that result in vasodilatation and antiplatelet aggregation and have antiproliferative effects. There is little evidence supporting the use of pulmonary arterial hypertension therapies in patients with POPH, and it is known that in this type of pulmonary hypertension there is a delay in the initiation of treatment and more tendency to monotherapy.^2^ In fact, the first randomized control trial in POPH (PORTICO), showed that macitentan gets a 35% reduction of PVR versus placebo and increases cardiac index, with no hepatic safety concerns.^3^ Macitentan, a dual endothelin receptor antagonist, developed by modifying the structure of bosentan to increase efficacy and safety, demonstrated in the REPAIR study, by cardiac magnetic resonance imaging, an improvement in the structure and functionality of the RV, achieving an average increase in right ventricular stroke volume at week 26 with respect to the baseline value of 12 mL, a 10.6% increase in right ventricular ejection fraction, as well as a 38% decrease in PVR.^5^ We believe that the patient improved with the new treatment not only because a decrease in PVR, also an improvement in RV function and structure was obtained. POPH is not an indication by itself for liver transplantation, being this procedure reserved for very selected cases.

In conclusion, we report a case of POPH successfully treated with combined medical treatment (tadalafil plus macitentan) where comorbidities have also been adequately managed.

## AUTHOR CONTRIBUTIONS

María Hernández, Violeta Esteban, Paloma Ruiz and Ignacio Boira drafted the manuscript. Phillip Wickmann, José Norberto Sancho‐Chust and Joan Gil revised the manuscript. All authors approved the final manuscript.

## CONFLICT OF INTEREST STATEMENT

None declared.

## ETHICS STATEMENT

The authors declare that appropriate written informed consent was obtained for the publication of this manuscript and accompanying images.

## Data Availability

Research data are not shared.
